# Investigating the influence of mineral content changes on mechanical properties through ligament insertion

**DOI:** 10.3389/fragi.2025.1556577

**Published:** 2025-07-07

**Authors:** Afif Gouissem, Fadi Alkhatib, Malek Adouni

**Affiliations:** ^1^ Mechanical Engineering Department, Australian University, Mishref, Kuwait; ^2^ Biomedical and Instrumentation Engineering, Abdullah Al Salem University, Khalidiya, Kuwait

**Keywords:** ligament insertion, mineral content, mechanical properties, collagenous tissues, molecular Dynamics simulation

## Abstract

**Introduction:**

This study investigates the relationship between mineral content and mechanical properties in collagenous tissues using a mesoscopic model. Unlike previous studies that assumed uniform mineral distributions, our model mimics the impact of combined intrafibrillar and extrafibrillar progressive mineralization on the ligament insertion using a realistic mineral gradient. To our knowledge, this is the first study on a minerally graded region that combines both mineral phases within a mesoscopic Molecular Dynamics framework.

**Methods:**

A collagen fibril model is constructed, and Molecular Dynamics (MD) simulations are performed at five equidistant locations along the insertion to analyze the influence of mineralization on collagen fibrils. The model captures the real randomness in mineral cluster size and distribution, improving its accuracy.

**Results:**

Results show that while Young's modulus and ultimate tensile strain remain relatively unchanged, ultimate tensile strength, yield strain, and yield strength are significantly affected by the presence of the mineral content. These changes are mainly caused by the interatomic bonds that restrain the collagen molecular sliding within the fibril.

**Discussion:**

Clinically, this research sheds light on the mechanical role that the progressive mineral gradient plays in load transfer and stress distribution. It also lays the ground for exploring the effects of aging and other pathological conditions such as ectopic mineralization or calcific tendinopathy, which alter the natural mineral gradient and increase the risk of tissue failure.

## 1 Introduction

Most body organs include collagen, which makes up over 30% of the total protein content and is the main constituent of human connective tissue. More than 20 different forms of collagen have currently been recognized (Ricard-Blum, 2011), among which type I and II are the most abundant and are widely present in bones, ligaments, and tendons (Buckley et al., 2013; Adouni and Dhaher, 2016; Faisal et al., 2019).

Understanding the mechanical properties of connective tissues, especially tendons and ligaments, has always been a focus of biomechanics because of the crucial role these tissues play in controlling human movement. In this context, several studies have analyzed their mechanical properties to determine the key factors impacting their strength and ductility (Khatib et al., 2021; Adouni et al., 2021; Mixon et al., 2021). Although aging was long considered the main factor in tissue elasticity loss (Kwan et al., 2023; Narici and Maganaris, 2006; Kubo et al., 2022), several studies have shown that it is not age itself, but rather the changes associated with aging that are responsible for the tissue deterioration. These changes include increased enzymatic and non-enzymatic cross-link densities within collagen fibrils, as well as changes in mineral content (Niu et al., 2024; Kamml et al., 2024).

Ligament insertions are among the connective tissues that have been extensively studied (Cho and Kwak, 2020; Neumann et al., 1992; Adouni et al., 2023a; Adouni et al., 2023b; Adouni et al., 2020; Adouni et al., 2022a). Their role is to anchor ligaments to bones, ensure joint stability and alignment, guide joint movement, and prevent excessive or abnormal motion. The insertion points are specifically adapted to handle the stress and tension exerted during physical activities by optimizing the stress transfer between ligaments and bones. In fact, the gradual properties of the insertions (partially due to the gradual change in mineral content) help distribute forces more effectively, minimizing the risk of injury.

Previous studies employed different approaches to estimate the response of collagenous tissues (including ligaments, bones, and ligament insertions) under mechanical loading: experimental tests have been performed to extract stress-strain curves for these tissues (Svensson et al., 2010; Liu et al., 2016; Yamamoto, 2017; Yamamoto and Nakamura, 2017). These methods are limited in their ability to establish a clear relationship between macro-scale results and molecular mechanisms. Molecular Dynamics simulations, on the other hand, target the properties of the fibril itself without needing to derive them from the macro-properties of the tissue (Saitoh et al., 2020; Ndlovu et al., 2012), and can provide insights into the underlying mechanisms. However, they may lose accuracy in determining the macro-properties given the complexity of the multi-scale modeling that needs to be involved (Adouni et al., 2022b).

In this paper, we address the problem from a molecular perspective by modeling the presence of mineral content in the collagenous tissue in its intrafibrillar and extrafibrillar forms, by performing Molecular Dynamics simulations to establish some insights into the change of mechanical properties through the insertions, and to identify the role of mineralization in determining the strength of the collagenous tissues.

While several previous studies have explored the mechanical behavior of partially mineralized collagenous tissues either through large-scale experimental testing (Nair et al., 2014; Milazzo et al., 2020; Gautieri et al., 2011) or through molecular simulations (Haba et al., 2012; Wopenka and Pasteris, 2005; Finnilä et al., 2020), they often overlook the gradual nature of mineralization found in real ligament insertions. These studies assume homogeneous and simplified mineral distributions, which do not accurately capture the complex mechanical environment at the interface.

In contrast, our study uses a mesoscopic model that mimics the gradual change in mineral content throughout the ligament-to-bone transition. The model explicitly considers both intrafibrillar and extrafibrillar mineral phases and allows us to replicate the natural mineral gradient observed in biological tissues. This enables us to analyze the effects of mineralization on collagen fibrils in a more realistic manner and to offer perspectives on the effect of mineralization on tissue mechanics by bridging the molecular and tissue scales.

By capturing this level of structural detail, our model provides insights into the role of mineralization on collagenous tissues in both healthy and pathological conditions.

## 2 Methodology

The investigation of the properties change through the ligament insertion begins by constructing a “mineral-free” collagen fibril. Molecular Dynamics simulations will be performed by adding the appropriate mineral content based on the sample position through the insertion.

### 2.1 Mineral-free collagen fibril model

The collagen molecule is a triple helical protein structure that consists of three chains of amino acids. It has a diameter of about 1.6 nm and a length of about 300 nm (Orgel et al., 2006). The above-described molecule is replicated orthogonally to its principal axis in a quasi-hexagonal array where each group of 5 molecules packs together by exhibiting gap/overlap regions along the length of the fibril with an average periodicity of 67 nm (Gautieri et al., 2011; Sweeney et al., 2008; Watanabe-Nakayama et al., 2016).

A full molecular model of a single fibril contains a few million atoms, depending on its size. To address this length scale issue, the concept of the mesoscopic model was previously developed by Buehler (Buehler, 2006). It consists in simplifying the molecular geometry into a single chain of beads, where each bead represents multiple atoms in the full atomistic model (Depalle et al., 2015; Depalle et al., 2016; Malaspina et al., 2017; Buehler and Wong, 2007; Buehler, 2008). This approach allows Molecular Dynamics to reach time and length scales otherwise inaccessible by conventional molecular simulations.

In this work, we assume that molecules are hexagonally packed (with a lattice constant of 16.52 Å), forming a fibril with a 21.5 nm diameter and containing 151 molecules (32,918 beads). A gap length of 36 nm and an overlap length of 28.2 nm are used. Details of the geometrical parameters of the model are presented in Table 1.

**TABLE 1:** Mesoscopic structural and geometrical properties.

Parameter	Value
Molecule number of atoms	3,134 atoms
Molecule total mass	287 kDa
Number of beads per molecules	218 beads
Mass of each bead	1,316 Da/mol
Length along principal axis	3,011 Å
Gap	400 Å
Overlap	∼282 Å
D-period	∼682 Å
Length of fibril	3,410 Å
Hexagonal lattice constant	16.52 Å

The atomic interactions in the fibril are governed by an energy function consisting of three primary components (Equation 1):

- Interatomic Energy: modeled using Lennard-Jones (LJ) potential, which regulates radial cohesion between molecules (Equation 2).
- Bond Energy: modeled by a hyperelastic potential, which controls the axial deformation between adjacent beads within a molecule (Equation 3).
- Angular Energy: modeled by a harmonic potential, which ensures proper alignment of the beads (Equation 4).

$$E=E_{inter}+E_{bond}+E_{angle}$$ (1)

$$E_{inter}=4\varepsilon \left({\left(\frac{\sigma }{r}\right)}^{12}-{\left(\frac{\sigma }{r}\right)}^{6}\right)$$ (2)

$$F_{bond}=\frac{\partial E_{bond}}{\partial r}=\left\{\begin{array}{l} K_{T0}\left(r-r_{0}\right),r<r_{1}\\ K_{T1}\left(r-\bar{r_{1}}\right),r_{1}<r<r_{b}\\ 0,r>r_{b} \end{array}\right.$$ (3)

$$E_{angle}=K_{\Theta }{\left(\theta -\theta_{0}\right)}^{2}$$ (4)

where σ and ε represent respectively the characteristic distance and the minimum energy of the LJ potential, $K_{T0}$ and $K_{T1}$ are spring constants, $r_{1}$ is the distance at which the hyperelastic behavior of the bond is triggered, $r_{b}$ is the bond breaking distance, $\bar{r_{1}}$ is a constant calculated to ensure the continuity of the force field, $K_{\Theta }$ represents the bending strength, $\theta_{0}$ represents the equilibrium angle, and θ represents the actual angle between the three consecutive beads. Collagen force field parameters are summarized in Table 2.

**TABLE 2:** Collagen force field parameters.

Symbol	Parameter	Value
r_0_	Equilibrium distance	14.00 Å
r_1_	Critical hyperelastic distance	18.20 Å
r_b_	Bond breaking distance	21.00 Å
K_T0_	Stretching strength constant	17.13 kcal/mol
K_T1_	Stretching strength constant	97.66 kcal/mol
ε	Lennard Jones energy	6.87 kcal/mol
σ	Lennard Jones effective radius	14.72 Å
$\theta_{0}$	Equilibrium bending angle	164–180°
$K_{\Theta }$	Equilibrium bending constant	14.98 kcal/mol/rad^2^

### 2.2 Mineralized collagen fibril model

The above-described model does not take into consideration the presence of minerals in the collagenous matrix. As that might be a valid assumption in several tissues where the actual mineral content is low, mineral crystallization must be considered in ligament insertions.

Genin et al. (2009) have measured the Mineral Volume Fraction (MVF) and have proven that it ranges between 9.4% on the ligament side to 65.2% on the bone side and that it changes linearly through the insertion. In this work, we will examine the whole range by considering five equidistant data points corresponding to a relative position of $x_{1}=0;\;x_{2}=0.25;\;x_{3}=0.50;\;x_{4}=0.75;\;x_{5}=1$ where x=0 corresponds to the position at the ligament limit, and x=1 corresponds to the position at the bone limit. The corresponding mineral volume fractions are as follows: $M{VF}_{1}=9.4\%;\;MVF_{2}=22.3\%;\;MVF_{3}=37.3\%;\;MVF_{4}=51.3\%;\;MVF_{5}=65.2\%.$ We will then investigate if the linearity of the mineral content fraction is translated into a linear change in the different collagen properties.

Starting from the mineral-free collagenous model, different approaches can be followed to insert the mineral content. In fact, the conventional view of biomineralization relies on a crystallization pathway starting from an amorphous phase in the gap regions and infiltrating through the collagen microfibrils. The amorphous phase then crystallizes into minerals when it moves to its final mineralization position. Challenging this theory, a newer standpoint of biomineralization suggests that, at an early stage of the biomineralization process, HAp (Hydroxyapatite) nanocrystals, which is the dominant mineral phase in collagenous tissue, can be deposited within the gap zone of collagen fibrils (forming intrafibrillar mineralization). At a later stage, they can precipitate on the surface of collagen fibrils, forming extrafibrillar mineralization. These processes lead to collagen fibrils containing both intrafibrillar and extrafibrillar mineral phases (Buehler and Wong, 2007; Buehler, 2008; Genin et al., 2009). Regardless of the mineralization pathway, the two approaches intersect in the fact that both intrafibrillar and extrafibrillar minerals are present within the tissue. While the larger part of the bone and ligament mineral is present in an extrafibrillar form ( ≈75% ), the rest is intrafibrillar.

While transitioning from amorphous to crystalline state, the HAp crystallizes into ellipsoid-like shapes in the gap region to fill the voids between the collagen chains, then nucleates into platelet-shaped blocks arranged in periodical arrays and oriented such that the long axis of the crystals (c-axis) is parallel to the long axis of the collagen. Around the fibril, the extrafibrillar crystals are also mostly aligned with the collagen fibrils (Genin et al., 2009), though local differences in alignment were observed for the disordered regions of bone (Tao et al., 2015). Their typical size ranges from 2 to 7 nm in thickness, 10–80 nm in width and 15–200 nm in length depending on the fibril diameter, the age and the location of the tissue.

Therefore, a single HAp platelet includes between 15,000 and a few hundred thousand atoms, which renders the full atomistic modeling inaccessible to conventional MD simulations. To remedy this issue, we use the same mesoscopic approach in defining HAp structure by abbreviating each cluster of atoms into a single bead, and then by defining the inter-atomic interactions between the HAp-HAp beads, and the HAp-Col beads. Details on the interatomic parameters were developed by Depalle et al. (2016) and are summarized in Table 3.

**TABLE 3:** HAp force field parameters.

Symbol	Parameter	Value
r_0_	Equilibrium distance	10.00 Å
r_1_	Critical hyperelastic distance	12.00 Å
r_b_	Bond breaking distance	14.89 Å
K_T0_	Stretching strength constant	200.00 kcal/mol
K_T1_	Stretching strength constant	418.40 kcal/mol
$\varepsilon_{Hap-Hap}$	Lennard Jones energy	137.1 kcal/mol
$\sigma_{Hap-Hap}$	Lennard Jones effective diameter	9.88 Å
$\varepsilon_{col-Hap}$	Lennard Jones energy	106.7 kcal/mol
$\sigma_{col-Hap}$	Lennard Jones effective diameter	10.28 Å

In the current model, we introduce the mineral content in three different ways.

- Intrafibrillar Mineral crystals: ellipsoids of HAp generated in the gap regions between the collagen molecules terminals (Long diameter: Φ_L_ = 2–34 nm to match the gap length, short diameter: Φ_S_ = 2–4 nm to match the gap thickness).
- Intrafibrillar Mineral platelets: the platelets are present in the (1,000) plane of the HCP collagenous structure as suggested by several studies (Tao et al., 2015). As our collagen model is on the lower end of the collagen fibril size spectrum, which can reach 500 nm in diameter (Versus 20 nm in our model), we use the following dimensions: (thickness: 2–4 nm x width: 5–10 nm x length: 5–40 nm).
- Extrafibrillar Mineral platelets: cylindrical shell blocks surrounding the collagen fibril (thickness: 2–4 nm x width: 0–40 nm x length: 15–100 nm).

All minerals were generated in random positions inside and around the collagen fibril one by one until the required mineral content was reached.

**FIGURE 1:**
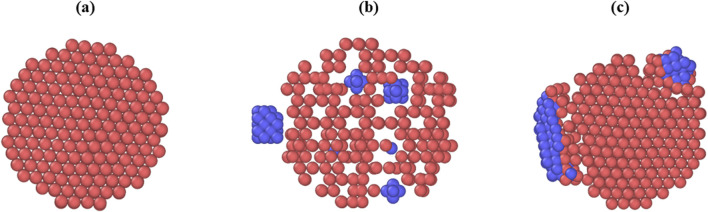
Snapshots of sections through the collagen fibril along the (0001) plane, captured before relaxation or equilibration. Collagen is shown in red, mineral inclusions are shown in blue. **(a)** Fibril with no mineral inclusions. **(b)** Fibril with one extrafibrillar inclusion positioned laterally and multiple intrafibrillar ellipsoidal inclusions embedded within the fibril core. **(c)** Fibril containing two extrafibrillar mineral clusters of different sizes.

**FIGURE 2:**
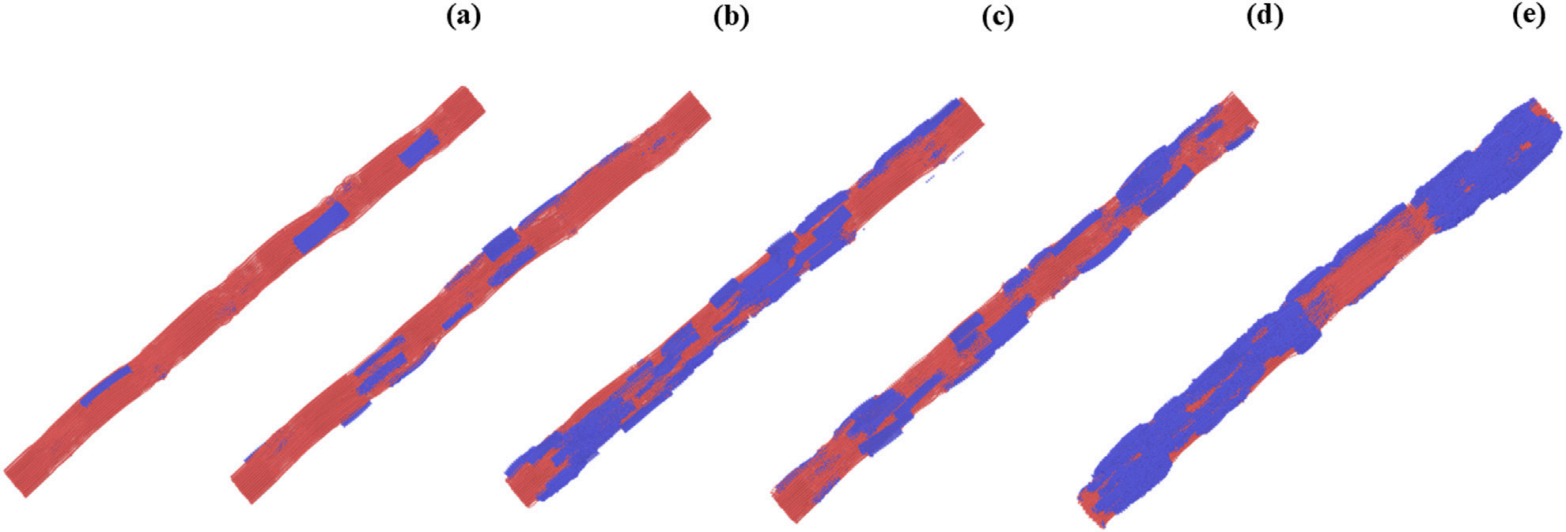
Snapshots of collagen fibrils modeled at different mineral volume fractions (MVF) and representing progressive mineralization across the ligament insertion. Collagen matrix is shown in red; mineral content (hydroxyapatite) is shown in blue. **(a)** Fibril at the ligament side (MVF = 9.4%). **(b–d)** Intermediate stages of mineralization at MVF = 22.3%, 37.3%, and 51.3%, respectively. **(e)** Fibril at the bone side (MVF = 65.2%).

Snapshots of the different sections of the mineralized fibril as well as snapshots of the full collagen fibril at different mineralization levels are shown in Figures 1, 2.

### 2.3 Simulation parameters

The fibril model was generated using MATLAB (2010), by averaging the geometric positions of the atoms in the 3HR2 PDB entry and replicating the molecule in the radial directions.

All MD simulations were performed using LAMMPS Molecular Dynamics software (Plimpton, 1995). A 10 fs time step was used. The fibril was relaxed at 300 K for 1 ns using NPT, then NVT to release the residual stress. To model the tensile deformation of the fiber, an axial velocity constraint was imposed on both ends of the fibril. Periodic boundary conditions were maintained in the longitudinal direction to mitigate any surface energy effects. Virial stresses and fibril volume were then used to compute the stress-strain curves. The visualization of the results was performed using the OVITO package (Stukowski, 2010).

### 2.4 Model verification

**FIGURE 3:**
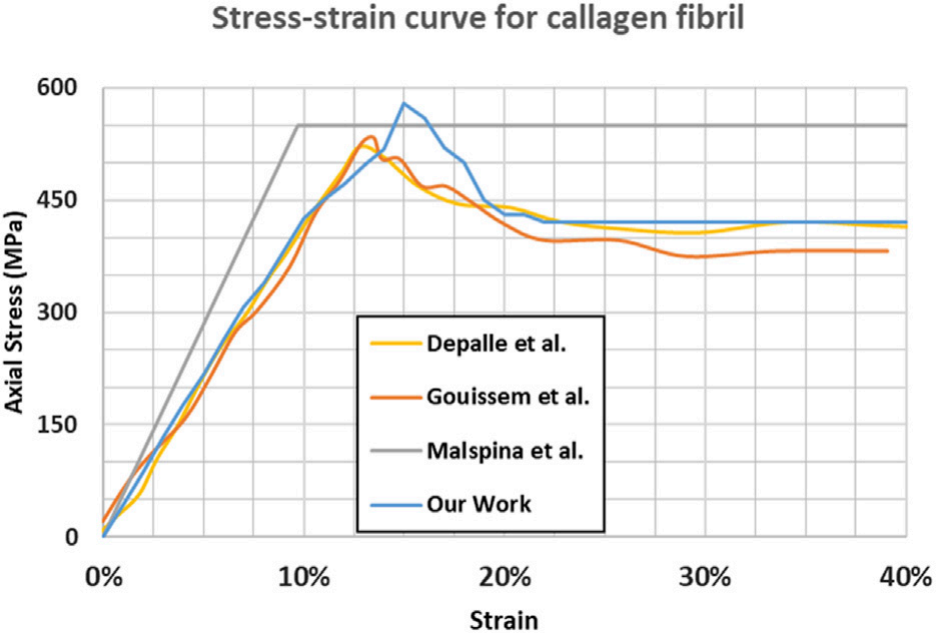
Stress-strain response for mineral-free collagen fibril.

The mineral-free collagen stress-strain curve (shown in Figure 3) aligns with the findings of several previous studies (Depalle et al., 2015; Malaspina et al., 2017; Gouissem et al., 2022) in terms of the main mechanical properties of the tissue. Figure 3 shows that for the absence of minerals, the differences between different studies are minor.

For instance, our Young’s modulus and ultimate tensile strength (4.68 GPa and 0.61 GPa respectively), are consistent with the findings in Depalle et al. (2015) (4.45 GPa and 0.55 GPa respectively).

We suggest that the minor differences between our model and the other studies are caused by several differences in the initial model geometry or the strain rate used:

- A gap/D-period ratio of 0.54 was used in this work versus 0.60 in other studies
- A total of 151 molecules were used versus 155 molecules in Depalle et al. (2015)
- No change in direction between the overlap and gap region is considered.

## 3 Results and discussion

Due to the randomness in numerous aspects of the collagenous tissue structure, several geometrical parameters are left intentionally variable in our model to mimic as closely as possible the real variations in the tissue (the position and the size of the mineral). Even though the mineral fraction and the proportions of the different crystallization forms are fixed within the model, the uncertainty in the size and location of each mineral cluster causes the stress distribution in the collagen to change.

**FIGURE 4:**
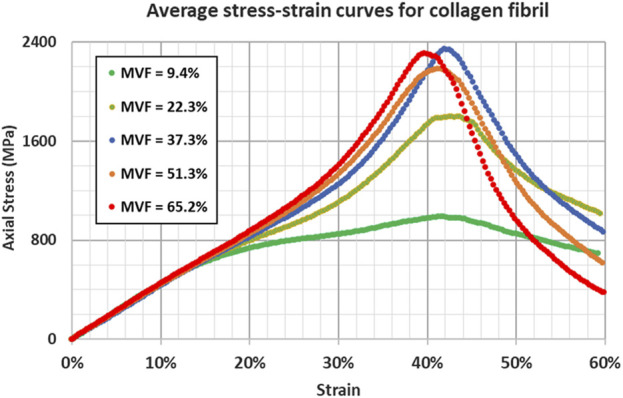
Stress-strain response for collagen fibril at different mineral volume fractions showing the influence of increasing the mineral content through the insertion.

Therefore, different simulations for the same mineral content fraction result in different stress-strain curves and, thus, different properties. Since the objective of the study is to analyze the effect of the mineral content, randomness (although realistic) can lead to misinterpreting the results. To mitigate this issue, we choose to perform five simulations for each data point. The averaged parameters are retained. Average stress-strain curves for different mineral content fractions are shown in Figure 4.

**FIGURE 5:**
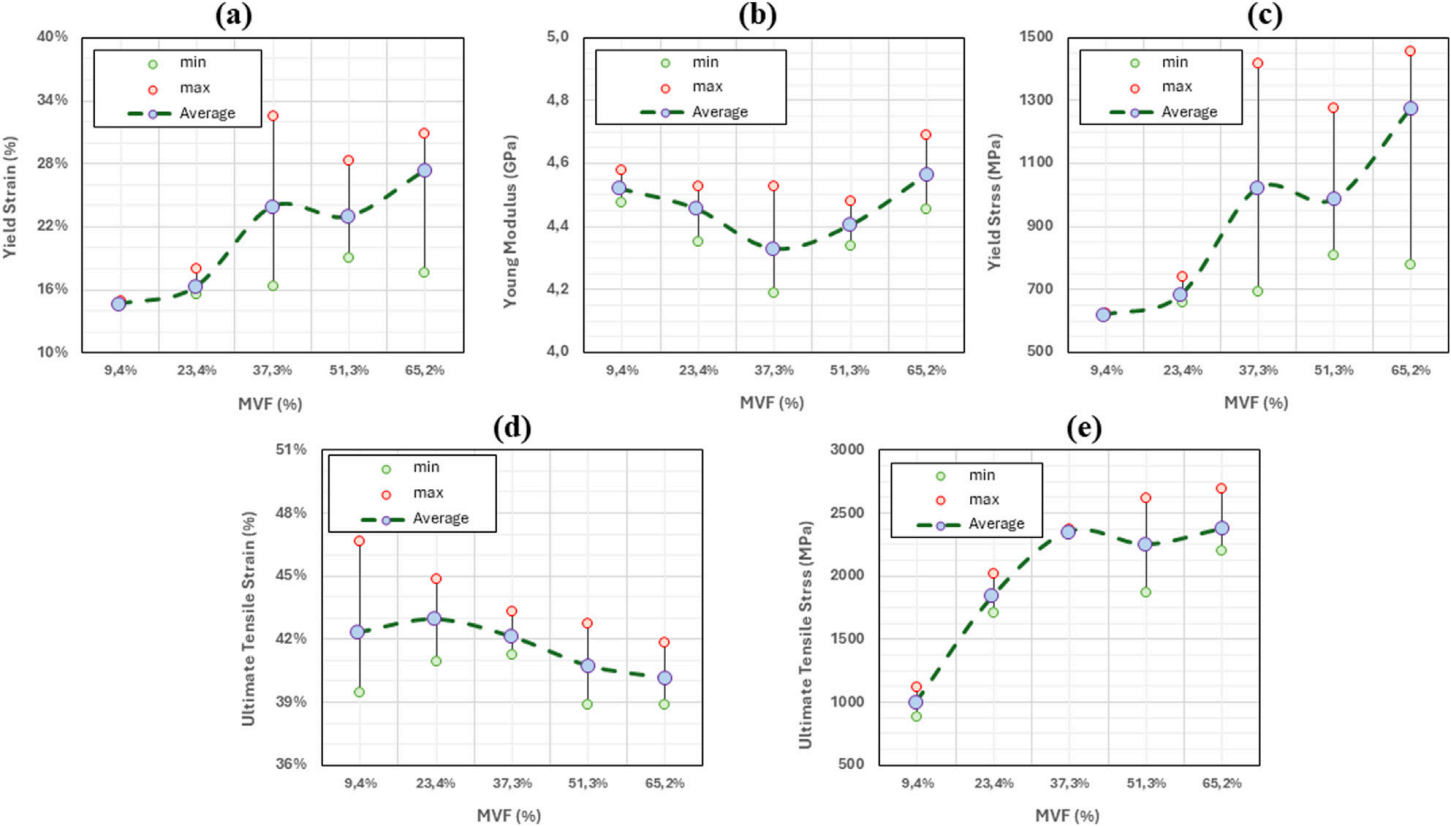
Variation of key mechanical properties of collagen fibrils as function of mineral volume fraction. For each MVF level, the minimum, maximum, and average values are reported based on five data points. Green circles indicate minimum values, red circles indicate maximum values, and purple-filled circles represent the average. The dotted gray line connects the average values to illustrate the overall trend. **(a)** Young’s modulus. **(b)** Yield Stress. **(c)** Yield Strain. **(d)** Ultimate Tensile Strain. **(e)** Ultimate Tensile Stress.

As mentioned in the methodology section, each curve represents an average of five different simulations. The detailed stress-strain curves for the different simulations are shown in Figure 5; Supplementary Figures S1–S5.

The different properties (Young’s modulus, yield stress, yield strain, ultimate tensile stress, and ultimate tensile strain) for each mineral content are also calculated and summarized in Table 4.

**TABLE 4:** Mechanical properties for different Mineral content fractions.

x	MVF	Young’s modulus (MPa)^a^	Yield strain^b^	Yield stress (MPa)	Ultimate tensile strain	Ultimate tensile stress (MPa)
0	9.4%	4,522	14.68%	619	42.33%	1,003
0.25	22.3%	4,458	16.30%	682	42.98%	1841
0.50	37.3%	4,331	23.96%	1,021	42.15%	2,355
0.75	51.3%	4,405	23.03%	987	40.61%	2,253
1	65.2%	4,566	27.42%	1,267	40.12%	2,378

^a^ Young’s modulus is calculated between 0% and 10% strain.

^b^ Yield point is calculated using 1% offset.

The variations of the mechanical properties with respect to the mineral content (or the position) are shown in Figure 5.

By analyzing the stress-strain curves and the graphs above, several interpretations can be drawn:

As additional minerals are added to the collagen matrix, we observe in Figure 5a that the yield strain remains unchanged between x = 0 and x = 0.25 (around 14%–15%). In fact, the yield point (characterized by the onset of molecules sliding) is reached when the shear radial Lennard Jones forces between the beads are no longer able to stop the longitudinal displacement of the molecules, causing relative sliding. The presence of the minerals provides an additional lateral force that holds the molecules in the radial direction, which increases the force required to cause the sliding. Based on this mechanism, one should expect an increase in the yield strain. However, since the fibril is constituted of a few hundred molecules, the presence of mineral clusters with lower concentrations does not affect all the molecules, but rather only the molecules to which they are attached. Consequently, some of the molecules will still slide as if no mineral content is present at that location, and the onset of sliding (the yield strain) remains unchanged. At higher concentrations of the minerals, the number of molecules that the HAp clusters are blocking increases. At our maximum mineral fraction, in the typical 15%–20% range, the material does not yield, indicating that no sliding is occurring. The values of yield strain presented for some of the curves at MVF = 62.3% correspond to the lift of the stress-strain curve from the elastic curve and not its drop, meaning that the collagen fibril reaches the work hardening phase corresponding to the activation of the hyperelastic properties of the collagen beads before any of the sliding occurs. Although we cannot determine for certain a specific concentration threshold where this phenomenon happens, as it will depend on the position and size of the minerals, which are randomly generated, we note that this specific mechanism occurred in four out of the five simulations at MVF = 62.3%, two of five at MVF = 51.25%, three out of five at MVF = 37.3%, and zero out of five at MVF = 9.4% and 23.35%. This explains the larger standard deviation we have for the higher mineral concentrations. We also observe that yield strains at the lower end of the MVFs are slightly higher than what was reported in the literature (Adouni et al., 2023b; Depalle et al., 2016; Gouissem et al., 2022). This is because we use 1% offset instead of 0.2% offset to determine the yield. This approach was adopted due to the fluctuation of the MD stresses that can reach a few megapascals which can erroneously suggest yield.

As detailed in our previous work (Khatib et al., 2021), the Young’s modulus of the collagen fibrils merely depends on the energy barriers defined by the collagen force field, and by the parameters of the elastic constants in the mesoscopic model. The presence (or absence) of minerals, or any additional forces that tend to block the sliding of the molecule, will only affect the strain at which the hyperelastic properties of the collagen are activated, but not the actual value of the Young’s modulus. The average Young’s modulus is 4.45 ± 0.12 GPa (2.6% variations) proving that the stiffness of the collagen does not change as shown in Figure 5b.

As the Young’s modulus is invariable to the mineral content fraction, the yield stress trend will follow the same trend as the yield strain explained above. Figure 5c shows very similar values at x = 0 and x = 0.25 with a very small standard deviation. At x = 0.5 and above, the yield stress shows large variations, showing values as low as 615 MPa (for models where actual yield occurred), and values as high as 1,500 MPa, representing the data points at higher yield strains where the yield is defined as the lift from the elastic curve, which represents the strain hardening of the collagen tissue.

The ultimate tensile point represents the location where the collagen molecules start sliding collectively after the lateral bonds are broken. In this context, the lateral bonds correspond to the force field linking collagen beads to mineral beads. Therefore, the ultimate tensile strain does not depend on the concentration but rather on the type and strength of the bonds. The higher the strength, the more elongation can be obtained before. Consequently, the ultimate tensile strain does not change with changing the mineral content as illustrated in Figure 5d where ultimate tensile strains range from 40% to 43% regardless of the MVF. In fact, the ultimate tensile strain is reached when every single molecule starts sliding and stress can no longer be accumulated on the bonds linking the mineral to the collagen beads. Two possible scenarios can occur: If the HAp-Col bond is stronger than the Col-Col bond, then the molecule itself will break in two, otherwise the bond will break first, and the molecule will slide. Either way, additional mineral content will cause higher stress accumulation (higher ultimate stress), but each of the bonds will break at the exact same point, given a relatively small strain rate.

In this context, the energy stored by the HAp-Col bond is 137.1 kcal/mol, while the energy that can be stored in a Col-Col bead bond is 613.7 kcal/mol, meaning that the adhesive forces from the mineral are not sufficient to break the collagen bonds, and the sliding will occur. The extra accumulation of stress in the HAp-Col bonds is reflected in the ultimate tensile strength as shown in Figure 5e.

While our model emphasizes the impact of mineral content, it should be noted that both enzymatic and non-enzymatic cross-links are important for the biomechanics of collagenous tissues. Enzymatic cross-links enhance the stiffness and tensile strength of collagen fibrils by covalently bonding collagen molecules (Fratzl and Weinkamer, 2007). This is captured in previous studies by a parameter β, which represents the ratio of cross-linked terminal sites. Several studies have demonstrated that increasing β primarily affects the ultimate tensile strength by covalently restraining the sliding of collagen molecules (Buehler, 2008). Given the heterogeneous nature, strength, and spatial distribution of cross-links, especially those of non-enzymatic origin, accurately modeling them is challenging. In this study, we chose to adopt a cross-link-free approach to isolate the effect of mineral content on the mechanical properties of the fibril.

Structurally, cross-links contribute to restricting the sliding of collagen molecules the same way that mineral clusters do. As a result, all stress-strain curves will shift upward, leading to higher values across mechanical properties. However, we believe that the trends described in the previous discussion would remain unchanged, even though absolute values would differ.

In summary, the progressive increase in mineral content along the ligament-to-bone interface has several implications for the mechanical properties of the tissue. However, it is more than a structural or mechanical feature, as it also plays a key biological and clinical role. This gradual transition ensures a smooth distribution of loads between the ligament and the bone, thus reducing stress concentrations (Apostolakos et al., 2014; Gracey et al., 2020).

Beyond the mechanical aspect, the observed mineral gradient across the insertion also plays a crucial biological role in the tissue function (Lei et al., 2021). As the tissue gradually changes from soft ligament to hard bone, it creates a local environment that helps define how several tissue cells behave. This includes influencing how easily certain cells, such as tenocytes, chondrocytes, and osteoblasts grow across each segment of the insertion (Benjamin et al., 2002). These natural variations help maintain the structure of the tissue and contribute to tissue repair when needed.

Because of aging, injury, or disease, the natural mineral gradient can be disturbed, thus compromising these biological processes. For instance, studies have shown that ectopic mineralization or mineral calcification, which are characterized by abnormal mineral depositions, can potentially lead to reduced tenocyte generation and therefore to irregular collagen alignment and compromised mechanical resilience.

From a clinical perspective, the change in mineral content across the insertion zone has significant implications, particularly when considering ligament or tendon repairs in both young and aging populations (Lu and Thomopoulos, 2013; Thomopoulos et al., 2003). Our results demonstrate that because of the linear mineral volume fraction increase from the ligament side to the bone side, the mechanical behavior of collagen fibrils changes along the insertion. In healthy tissues, this gradual transition ensures smooth load transfer and minimizes stress concentrations at the interface.

With aging, the smooth mineralization transition at the ligament-to-bone insertion can become highly disorganized (Thomopoulos et al., 2010). Several studies have shown that aged tissues often exhibit ectopic mineralization, where hydroxyapatite deposits appear in high concentrations in typical “low mineral” zones closer to the ligament side. This abnormal mineral buildup can disrupt the mechanical properties and alter the transition that normally protects the tissue from high stress, making it more susceptible to damage.

In the context of clinical repair, specifically in older patients, the disrupted mineralization pattern has practical consequences (Zelzer et al., 2014). In ligament and tendon reconstruction procedures (such as ACL or rotator cuff repairs), the natural gradient shown in our model, which correlates with progressive changes in collagen fibril mechanics, is rarely restored during surgical procedures. Instead, grafts are fixed into bone tunnels without a mineral transitional zone. This leads to higher stress concentrations at the interface, which can, over time, result in poor osseointegration and higher failure rates.

## 4 Conclusion

While our present study is, to our knowledge, the first to model the mineral content using a mesoscopic approach that combines the intrafibrillar and the extrafibrillar contents, and to perform Molecular Dynamics simulations on a fully mineralized fibril, some assumptions in our methodology may be debatable without altering the major insights of our study. Considering that the structure, the orientation, and the percentage of the collagen fibrils change from the ligament to the bone, the first question that arises is the uniformity of the macro-structure of the collagenous tissue through the ligament insertion. We are abbreviating the changes into weight percentage change through the insertion without looking further for additional changes. In fact, we deem this approach acceptable in our context since we are only showing the stress-strain behavior of single collagen fibrils. Changes in mineral content in the fibril itself and in its surrounding matrix are taken into consideration. If stress-strain behavior on the macro scale is to be extracted, then the change in orientation and structure should be taken into consideration.

Another important consideration is the absence of cross-links in our current model. Although we do acknowledge that both enzymatic and non-enzymatic cross-links play a crucial role in altering tissue properties, we focus on the mineral fraction as the main influencing factor in this context. In fact, the cross-links will play the role of an offset by altering all data points in the same direction, without affecting any of the conclusions drawn. For this reason, we assume a cross-link-free mineralized collagen fibril. Future simulations can add more densities and types of crosslinks to the current model to see how they interact with each other and with mineralization. This is especially important in aged or pathological tissues where crosslinking and mineralization co-evolve.

In conclusion, the study explored the influence of mineral content on the mechanical properties of collagen fibrils using a mesoscopic model. By incorporating randomness in mineral cluster size and distribution, the model realistically captured the variability observed in natural tissues without compromising the reliability of the results.

Key findings show that while the Young’s modulus and ultimate tensile strain are largely unaffected by changes in mineral content, yield strain and yield stress exhibit significant changes. Higher mineral fractions increase the lateral forces restraining molecular sliding, resulting in greater yield strain and stress. The ultimate tensile stress also increases with mineral content, reflecting increased stress accumulation before bond failure. These mechanical changes have implications on the tissue behavior, particularly under physiological and pathological conditions.

It is also important to highlight a potential link between mineral content and aging. As individuals age, the mineral fraction in collagen tissues often increases, leading to altered mechanical properties. Higher mineralization may contribute to a decrease in tissue flexibility and resilience, potentially predisposing tissues to conditions such as ligament tears, reduced joint mobility, and other degenerative issues. The findings underscore the importance of understanding these changes to develop preventive or therapeutic strategies for age-related connective tissue disorders.

## Data Availability

The original contributions presented in the study are included in the article/Supplementary Material, further inquiries can be directed to the corresponding authors.
