# Supplementary material for: Investigating the influence of mineral content changes on mechanical properties through ligament insertion
Source: Front Aging. 2025 Jul 7;6:1556577. doi: 10.3389/fragi.2025.1556577 (PMC12277273; doi:10.3389/fragi.2025.1556577)
Supplement: Supplementary file 1 [file DataSheet1.pdf]

Supplementary Material

1 Supplementary Figures:

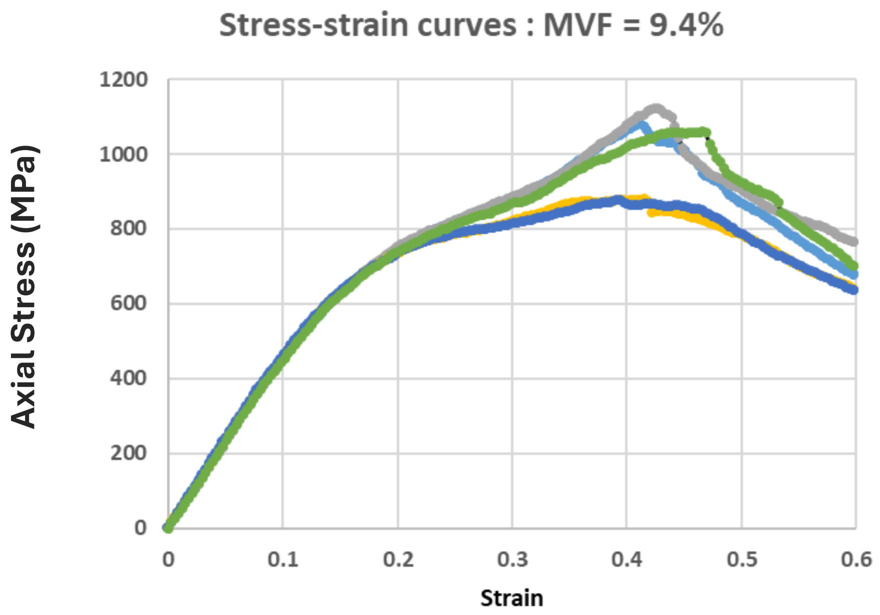

Supplementary Figure 1. Stress-strain field for collagen fibril for 9.4% mineral fractions.

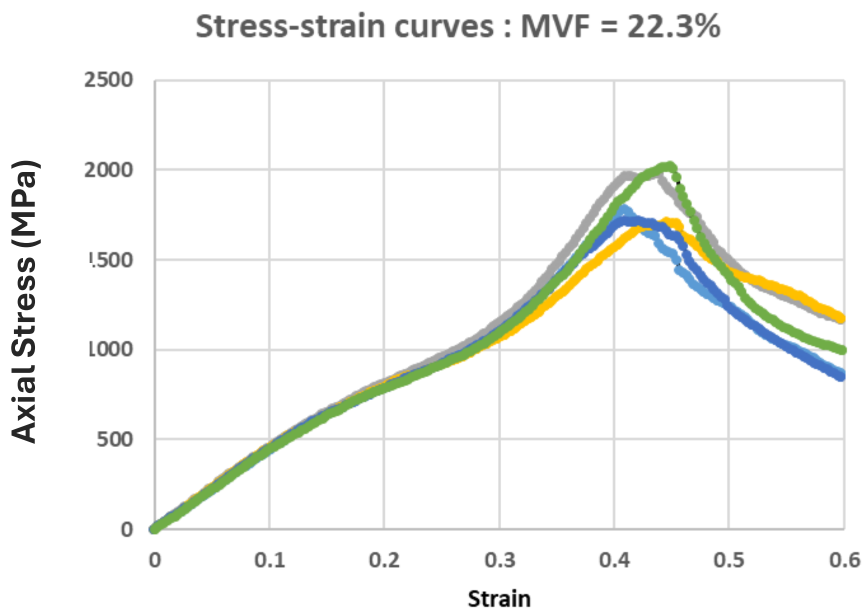

Supplementary Figure 2. Stress-strain field for collagen fibril for 22.3% mineral fractions.

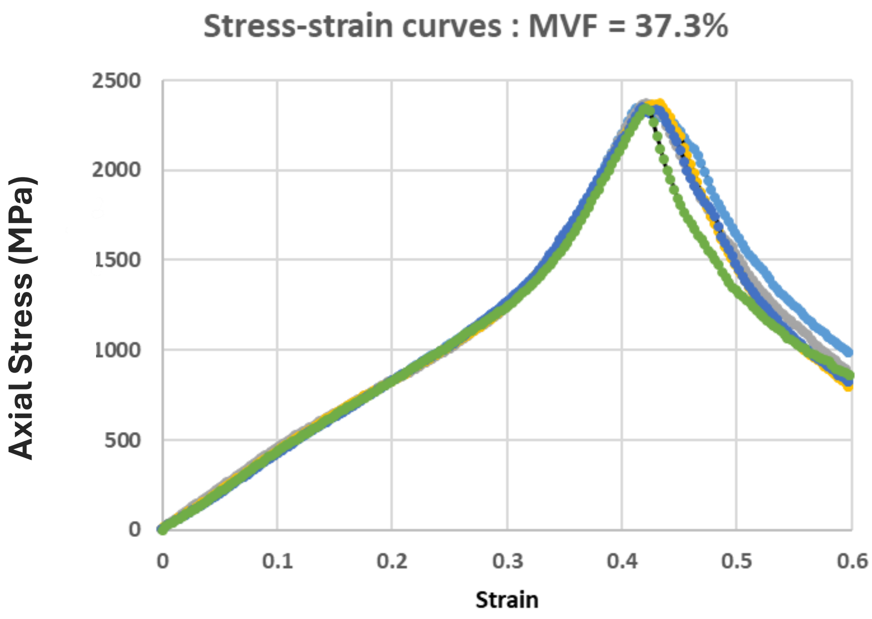

Supplementary Figure 3. Stress-strain field for collagen fibril for 37.3% mineral fractions.

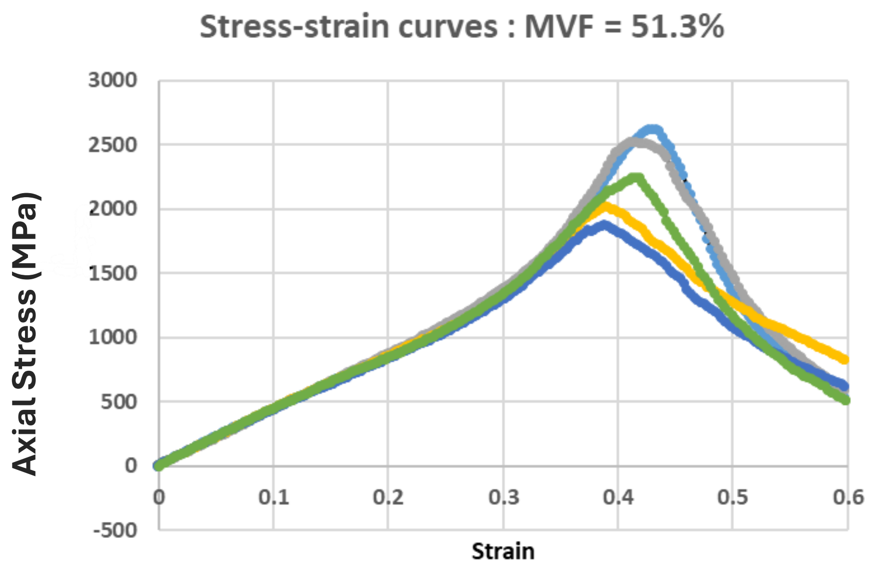

Supplementary Figure 4. Stress-strain field for collagen fibril for 51.3% mineral fractions.

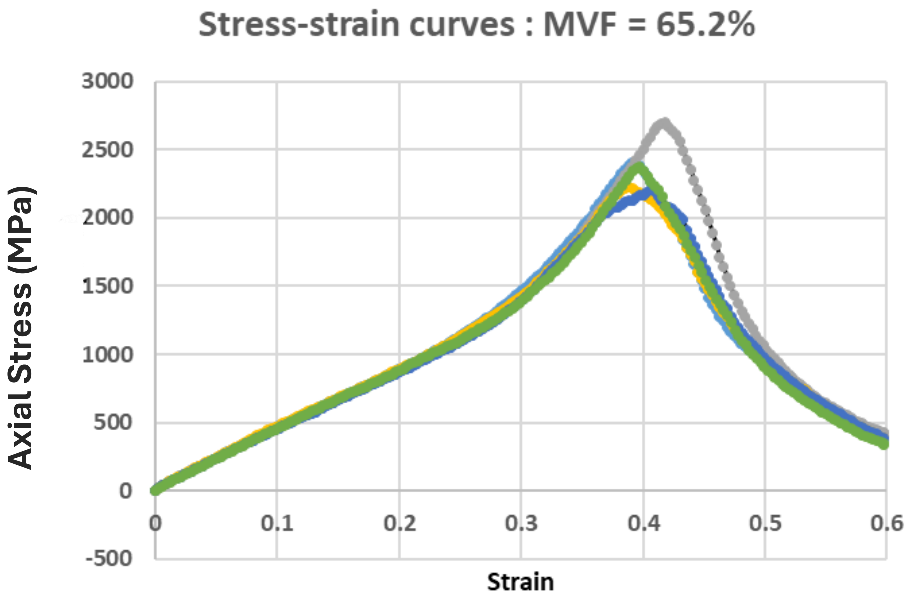

Supplementary Figure 5. Stress-strain field for collagen fibril for 65.2% mineral fractions.
